# Machine Learning Assessment of the Environmental Factors Contributing to Shade Adaptation in *Brassica juncea*

**DOI:** 10.3390/plants15050780

**Published:** 2026-03-03

**Authors:** Bae Young Choi, Eunji Bae, Ick-Hyun Jo, Jaewook Kim

**Affiliations:** 1 School of Liberal Arts and Sciences, Korea National University of Transportation, Chungju 27469, Republic of Korea; baeyoung@ut.ac.kr; 2 Forest Biomaterials Research Center, National Institute of Forest Science, Jinju 52817, Republic of Korea; gosorock@korea.ac.kr; 3 Department of Crop Science and Biotechnology, Dankook University, Cheonan 31116, Republic of Korea; intron@dankook.ac.kr; 4 Department of Biology Education, Korea National University of Education, Cheongju 28173, Republic of Korea

**Keywords:** *Brassica juncea*, shade avoidance syndrome, climate, artificial intelligence, random forest

## Abstract

*Brassica juncea* is a widely cultivated leafy vegetable species in Northeast Asia, including Korea, Japan, and China. Under shade conditions, *B. juncea* exhibits shade avoidance syndrome (SAS), which negatively impacts its market quality. However, *B. juncea* is cultivated in diverse climates worldwide, including regions with frequent foggy days, highlighting the need to understand its adaptation to shade conditions to improve cultivation quality. To investigate the relationship between SAS phenotypes and environmental factors, including daylength, precipitation, and temperature, we analyzed 30 clones and six commercial cultivars of *B. juncea*. After 7 days of growth, all six commercial cultivars exhibited a canonical SAS response, with hypocotyl length increasing by 3.25- to 5.18-fold under dim light compared to white light conditions. Among the 30 clones, shade responsiveness varied widely, with hypocotyl elongation ranging from 1.42- to 8.54-fold change. A simple correlation analysis revealed that environmental factors were not highly correlated with shade responsiveness due to their complex interactions. To address this, we applied six machine learning models and found that the random forest algorithm provided the most accurate predictions of environmental influences on hypocotyl length. Using this model, we identified daylength, precipitation, and temperature as key environmental factors contributing to SAS phenotypes in *B. juncea*. Our findings not only identify clones that can be cultivated under low-light conditions with reduced SAS effects but also establish a link between SAS phenotypes and natural environmental conditions. These insights provide a foundation for future breeding strategies to improve shade adaptation in *B. juncea*.

## 1. Introduction

*Brassica juncea*, commonly known as Korean green mustard or oriental mustard, can be subdivided into four groups: *integrifolia* (leaf mustard), *juncea* (seed mustard), *napiformis* (root mustard), and *tumida* (stem mustard). It is widely used as a leafy vegetable, a seed oil crop, and a key ingredient in fermented foods, with its economic value primarily determined by the quality of its leaves and seeds [1]. Although the species is believed to have originated from a single region in West Asia, it has since spread globally [2]. Over time, many wild-type clones have likely evolved to adapt to diverse environmental conditions, expanding the ecological range of *B. juncea*.

Variability in climate conditions can affect the crop development and plant physiological processes [3,4] and fruit quality across multiple regions [5,6]. The modeling approaches accessing crop responses to soil and environmental conditions have been raised in recent years [7,8]. Compared to process-based models (PBMs) that require more inputs, the mathematical and machine learning models work well in limited data conditions, as illustrated by Rafique et al. 2025 [9]. Studies on the effect of the climate conditions in leafy plants were also reported [10,11,12], not only for the effect but also the modeling approach was increased and valuable in leafy crops as well [13,14,15,16].

Shade avoidance syndrome (SAS) describes the adaptive response of sunny plants to shade, primarily regulated by phytochrome, which senses changes in the ratio of red to far-red light as an environmental cue [17]. Most of the research on SAS has focused on understanding its molecular mechanisms, particularly the phytochrome-PIF module (phytochrome-interacting factors, bHLH transcription factors) and their downstream signaling components, such as auxins [17]. These mechanisms drive succulent growth of seedlings, which exhibit elongated hypocotyls and petioles, reduced cotyledon size, and pale green cotyledons [18]. Shade responses have been correlated with both plant fitness in shaded environments and plant survival by altering vascular architecture within canopy gaps [17,19,20,21]. While some molecular mechanisms of SAS are understood, the responsible receptors and the major environmental factors affecting adaptation to shade conditions have not been fully explored.

Shade adaptation in plants has been studied in various facets. In some tree species, including *Abies alba*, *Taxus baccata*, and *Picea abies*, leaves in shaded conditions were found to accumulate lower levels of carotenoids compared to younger leaves exposed to sunlight [22]. In diverse wheat cultivars, shade conditions reduced photosynthetic activity in shade-sensitive cultivars but not in shade-resistant cultivars [23]. Evolutionary analyses have further supported these physiological findings; for example, in *Ardisia* plants, two protein-coding genes (*rbcL* and *rpoC2*) were positively selected during adaptation to shaded environments [24]. A similar result was observed in *Chrysosplenium sinicum*, where *Lhcb1s*, a component of the light-harvesting complex II, underwent positive selection throughout the adaptation process in shade conditions [25]. In addition to changes in photosynthesis, morphological adaptations to shade have been studied. In *Hippophae rhamnoides*, shade conditions resulted in increased leaf area and sharpened leaf inclination [26]. These shade-responsive morphological adaptations were strongly correlated with heat dissipation, suggesting a potential connection between shade adaption and thermal regulation [26].

When it comes to *B. juncea*, only a few studies have reported on its shade response. A recent study reported that simulating shade conditions by supplementing white light with additional far-red light resulted in enlarged leaves and accelerated the phase transition to the floral stage, with significantly reduced accumulation of anthocyanins and glucosinolates [27]. Additionally, some *MKK* genes have been suggested to be associated with shade responses [28]. However, no studies have investigated the physiology of SAS in *B. juncea* or its adaptation to shade conditions. In this study, we applied canopy conditions to *B. juncea* seedlings with various genetic backgrounds to analyze their SAS phenotypes, with a particular focus on hypocotyl length. Using ecotypes and natural cultivars from various regions, we aimed to identify key environmental factors influencing shade adaptation.

## 2. Results

### 2.1. Shade Avoidance Syndrome Phenotype in Six Commercial B. juncea Cultivars

**Figure 1:**
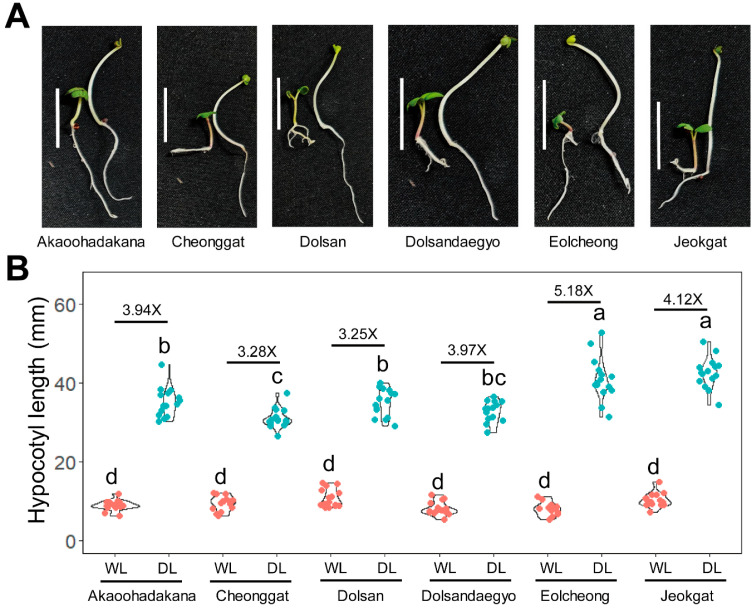
Shade stimulus induces hypocotyl elongation in *B. juncea* compared to white light conditions. (**A**) A Representative picture of 7-day-old seedlings from six commercial cultivars. The scale bar indicates 10 mm. The left seedling represents a seedling grown under white light (WL) for 7 days, while the right seedling represents a seedling grown under WT for 3 days and then transferred to dim light (DL) for 4 days. (**B**) Violin plot depicting hypocotyl length of seedlings under WL and DL conditions. Red dots indicate hypocotyl length under WL conditions, while cyan dots indicate hypocotyl length under DL conditions. The letters above the data points indicate statistical significance based on Tukey’s HSD test (*p* < 0.05). Average shade responsiveness is represented as the fold change in hypocotyl length relative to WL.

To analyze the shade-responsive phenotypes in *B. juncea*, we examined six commercially available cultivars: Akaoohadakana, Cheonggat, Dolsan, Dolsandaegyo, Eolcheong, and Jeokgat. Since *B. juncea* has a long history of cultivation and breeding, we selected these widely accessible cultivars to assess their physiological responses to shade conditions. Seeds from each cultivar were grown under white light conditions for 3 days and then either maintained under the same conditions for additional 4 days or transferred to dim light conditions (canopy conditions) for 4 days. All six cultivars showed increased hypocotyl length, and reduced cotyledon size under dim light conditions (Figure 1A). Focusing on hypocotyl length, all cultivars exhibited similar lengths under white light. However, their elongation responses under dim light conditions varied, with increases ranging from 3.25-fold to 5.18-fold compared to white light conditions (Figure 1B). Despite variations in the degree of response, all cultivars displayed the characteristic physiological traits associated with SAS (Figure 1).

### 2.2. B. juncea Landraces and Wild Types Had Wide Variations in Shade Responsiveness

**Figure 2:**
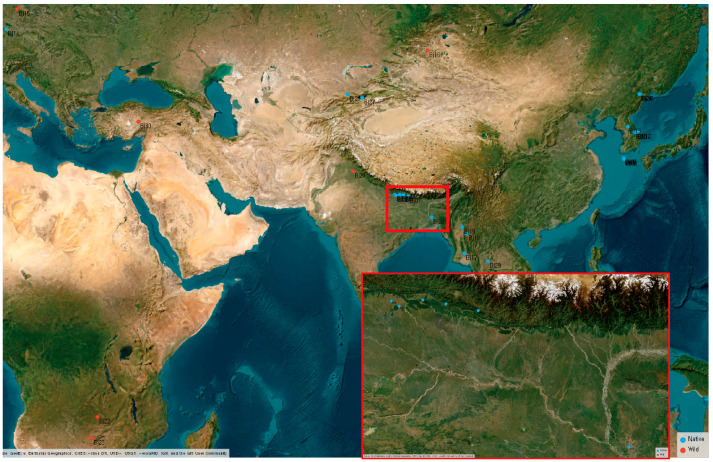
Geographical origins of analyzed wild-type or landrace *B. juncea* in this study. Blue dots indicate landraces, while red dots indicate wild types. Red box shows same region with different resolution.

**Figure 3:**
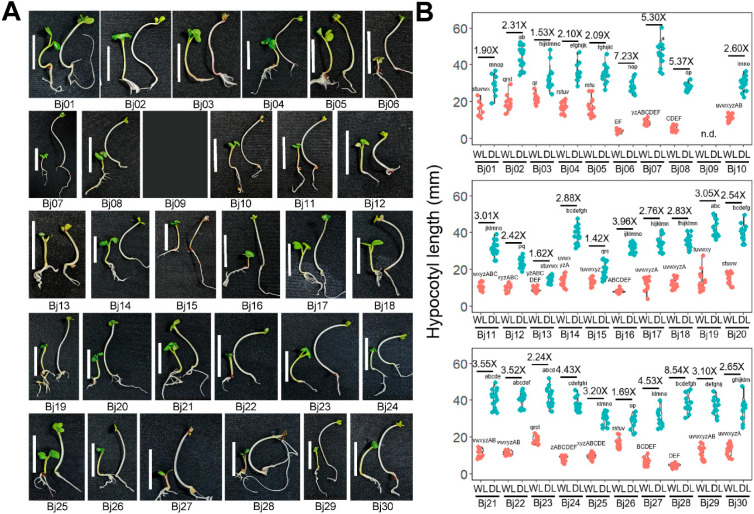
Shade avoidance syndrome analysis in 30 wild-type and landrace *B. juncea* clones. (**A**) A representative picture of 7-day-old seedlings from 30 clones. The scale bar indicates 10 mm. The left seedling represents a seedling grown under white light (WL) for 7 days, while the right seedling represents a seedling grown under WT for 3 days and then transferred to dim light (DL) for 4 days. Bj09 was omitted for bad germination rate. (**B**) Violin plot depicting hypocotyl length of seedlings under WL and DL conditions. Red dots indicate hypocotyl length under WL conditions, while cyan dots indicate hypocotyl length under DL conditions. The letters above the data points indicate statistical significance based on Tukey’s HSD test (*p* < 0.05). Although statistical tests were performed for all clones, graphs are shown separately for clarity. Average shade responsiveness is represented as the fold change in hypocotyl length relative to WL. n.d. indicates data not determined due to poor seed quality.

To study the contribution of environmental factors on shade adaptation in *B. juncea*, we collected 30 clones from of diverse geographic origins (Figure 2 and Table S1). Given the variability in their native environments, inferred from climate data of diverse origin sites (Table S2), these clones provided a valuable resource for assessing environmental variation in SAS responses. To evaluate their shade responsiveness, we performed the same physiological analysis used for assessing commercial cultivars in Figure 1. All clones germinated successfully and exhibited comparable growth rates, making them suitable for physiological assessment, except Bj09, which germinated too late for phenotype analysis. All the clones showed SAS responses, but the degree of responsiveness varied widely (Figure 3A). Some clones exhibited minimal shade-induced elongation, with hypocotyl lengths increasing by less than two-fold under dim light conditions, while a few were highly responsive, with elongation exceeding seven-fold increase (Figure 3B). Specifically, Bj01, Bj13, Bj15, and Bj26 showed weak SAS responses (Figure 3B). Among them, Bj01 and Bj26 had relatively long hypocotyls under white light conditions. Bj13 and Bj15 displayed a short hypocotyl length under white light and exhibited minimal hypocotyl elongation in response to dim light. In contrast, Bj06 and Bj28, which had very short hypocotyls under white light conditions, were highly responsive to shade stimulus, with significant hypocotyl elongation under dim light conditions (Figure 3B). Our physiological analysis of diverse *B. juncea* clones revealed considerable variation in shade responsiveness. These results suggest that the extent of SAS response may be influenced by the initial hypocotyl length under white light conditions.

### 2.3. Weak Correlations Between the Environmental Factors and Shade Responsiveness

**Figure 4:**
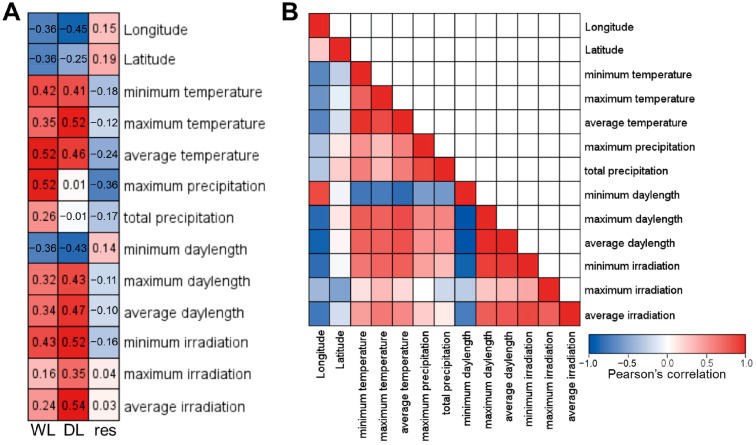
Simple Pearson’s correlation analysis between phenotypes and climate conditions of natural origin. (**A**) Heatmap depicting Pearson’s correlation coefficients between hypocotyl phenotypes and climate conditions of natural origin. WL indicates the hypocotyl length of seedlings grown under white light, DL indicates the hypocotyl length of seedlings grown under dim light, and res indicates shade responsiveness. All the data were normalized to a scale of 0 to 1 by simple proportion. The color key is provided in (**B**). (**B**) Heatmap depicting Pearson’s correlation coefficients among climate conditions of natural origins.

To reveal environmental factors correlated with variation in shade responsiveness among *B. juncea* clones, we calculated Pearson’s correlation coefficients (Figure 4A). Several environmental factors showed weak correlations with hypocotyl length under both white light and dim light conditions, with correlation coefficients exceeding 0.5 (Figure 4A). Specifically, average temperature and maximum precipitation exhibited weak positive correlations with hypocotyl length under white light conditions (Figure 4A). Under dim light conditions, maximum temperature, minimum irradiation, and average irradiation showed weak positive correlations with hypocotyl length (Figure 4A). However, no single environmental factor exhibited a correlation strong enough to explain shade responsiveness (Figure 4A). To address the complexity of environmental factors, we calculated Pearson’s correlation coefficients among all the climate data (Figure 4B). Many climate factors were highly correlated with each other, while some showed weak associations. While temperature and daylength data followed intuitive correlation patterns, irradiation data had complex relationships with other factors (Figure 4B). These findings indicate that environmental factors interact in intricate ways, making it difficult to pinpoint specific environmental factors influencing shade responsiveness using simple correlation analysis.

### 2.4. Random Forest Modeling to Identify the Key Environmental Factors Contributing the Shade Responsiveness

To address the complex contributions of environmental factors to shade responsiveness in *B. juncea*, we applied a machine learning approach. First, we evaluated six commonly used machine learning models to identify the best fit for our phenotype data (Figure S1A). Each model was trained using 80% of the phenotype data with corresponding environmental data, while the remaining 20% was used for prediction analysis (Figure S1B). RMSE was calculated to assess model accuracy, and the random forest model showed the best predictive performance (Figure S1B). To further validate the performance of random forest models, we examined the correlation between experimental and predicted data (Figure S2). All three models achieved strong predictive accuracy in estimating hypocotyl length under white light, dim light, and shade responsiveness, as evidenced by low RMSE values (Figure S2). These results indicated that the random forest pipeline is a suitable tool for predicting the hypocotyl length of *B. juncea* based on corresponding environmental data.

Next, we assessed the contribution of environmental factors in predicting hypocotyl length grown under white light and dim light conditions using the random forest models. Under white light conditions, latitude, minimum daylength, and maximum daylength were identified as the most important environmental factors for predicting hypocotyl length (Figure S3A,B). SHAP score analysis confirmed their strong contributions to the model (Figure S3C). Partial dependence plots revealed that a maximum daylength below 850 min and a minimum daylength above 620 min substantially increased predicted hypocotyl length, whereas latitudes between 30 and 40 substantially decreased it (Figure S3D–F). Under dim light conditions, average temperature, minimum temperature, total precipitation, and average irradiation were the most influential environmental factors in predicting hypocotyl length (Figure S4A,B). SHAP score analysis identified maximum precipitation, total precipitation, maximum irradiation, average irradiation, average temperature, and minimum temperature as key contributors (Figure S4C). Partial dependence plots showed that maximum precipitation below 300 mm and total precipitation below 1000 mm substantially increased predicted hypocotyl length (Figure S4D,E). Maximum irradiation around 210 kWh/m^2^ substantially decreased hypocotyl length (Figure S4F), while average irradiation above 120 or 140 kWh/m^2^ led to a rapid increase in predicted hypocotyl length (Figure S4G). Additionally, an average temperature of approximately 13 °C and a minimum temperature near −3 °C substantially reduced hypocotyl length (Figure S4H,I). These results suggest that minimum and maximum daylength played a major role in determining hypocotyl length under white light, while maximum precipitation and total precipitation were key factors under dim light conditions.

**Figure 5:**
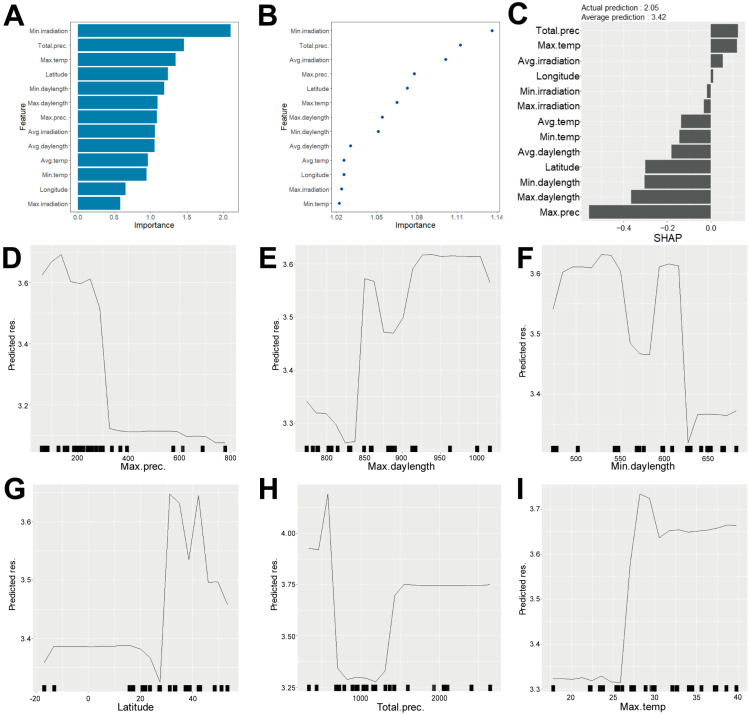
Random forest model analysis of shade responsiveness with corresponding climate data. A random forest model was constructed using phenotype data and climate conditions. (**A**) Bar graph representing feature importance in the model. (**B**) Dot graph representing feature importance determined by a permutation test. (**C**) Bar graph representing SHAP value. (**D**–**I**) Partial dependence plots of the six most influential features in predicting shade responsiveness. (**D**) Maximum precipitation vs. predicted shade responsiveness. (**E**) Maximum daylength vs. predicted responsiveness. (**F**) Minimum daylength vs. predicted responsiveness. (**G**) Latitude vs. predicted responsiveness. (**H**) Total precipitation vs. predicted responsiveness. (**I**) Maximum temperature vs. predicted responsiveness.

**Figure 6:**
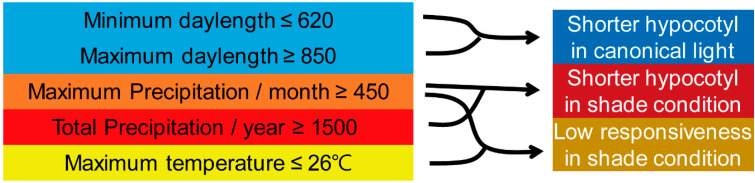
Proposed model illustrating the contribution of natural environmental conditions on shade-responsive hypocotyl phenotypes in *B. juncea*. The left boxes indicate the environmental factors identified as key factors in determining shade responsiveness and hypocotyl length, while the right boxes indicate the resulting physiological traits under different light conditions. The arrows in the middle show the predicted relationships between environmental factors and phenotypic traits, as revealed through the machine learning approach.

Finally, we evaluated the contribution of environmental factors in shade responsiveness using the random forest model. The most important environmental factors in this model were the average temperature, minimum irradiation, and total precipitation (Figure 5A,B). SHAP score analysis identified maximum precipitation, maximum daylength, minimum daylength, latitude, total precipitation, and maximum temperature as key contributors (Figure 5C). Partial dependence plots revealed that shade responsiveness was influenced by multiple environmental factors in a complex manner (Figure 5D–I). Specifically, maximum precipitation below 320 mm and maximum temperature above 26 °C substantially increased predicted shade responsiveness (Figure 5D,I). Meanwhile, daylength and latitude exhibited intricate contributions to shade responsiveness (Figure 5E–G). Additionally, total precipitation near 1000 mm substantially reduced shade responsiveness (Figure 5H). These findings suggested that maximum precipitation and maximum temperature were primary determinants of shade responsiveness in *B. juncea*. Taken together, our random forest modeling identified distinct environmental factors influencing hypocotyl length under different light conditions and shade responsiveness in *B. juncea* (Figure 6).

## 3. Discussion

### 3.1. Higher Precipitation Positively Correlated with the Number of Foggy Days Might Be Responsible for the Shade Adaptation

Our data showed that higher precipitation in maximum level per month and total precipitation per year are key environmental factors in regulating hypocotyl length under shade conditions (Figure S4). A notable fact is that precipitation is positively correlated with the number of rainy days and thus might have positive correlation with the foggy days [29,30]. In our data, total precipitation was moderately correlated with the minimum temperature and average temperature but not with maximum temperature in positive direction (Figure 4B). The minimum temperature of origins, with total precipitation of over 1500 mm ranged from 1.8 °C to 17.8 °C, and average temperature ranged from 14.2 °C to 26.5 °C (Table S2). The climate data suggested that hypocotyl length of the clones or landraces would have relatively shorter hypocotyl length in farm conditions. In that case, a higher chance of encountering the foggy days could be an environmental factor for shade adaptation.

### 3.2. Additional Factors Potentially Contributing to Shade Adaptation in B. juncea

Although we analyzed the contribution of climate factors to shade adaptation in *B. juncea* (Figure 6), other environmental factors not examined in this study may also play crucial roles in regulating shade responses. First, shade response includes decreased chlorophyll contents, reducing photosynthetic efficiency, which may act as a selective pressure on plants [21]. Although photosynthetic activity has been considered an important evolutionary driver, its role as a selective force in shade adaptation remains relatively unexplored [31,32]. Some studies suggest that shade conditions do not necessarily result in lower quantum yield in photosynthesis [33,34,35], implying an ambiguous role of photosynthetic activities in shade adaptation. The second environmental factor that may influence shade adaptation is soil enrichment, which has been shown to induce substantial physiological responses and interact with shade stimuli [36,37]. However, assessing soil enrichment as a natural selective pressure is challenging, as agricultural practices often involve compost application and green manuring applications [38,39,40]. Therefore, for landraces, soil enrichment cannot be considered a completely natural environmental factor. Third, the soil microbiome, which plays a crucial role in shaping global biodiversity, is another potential contributor to shade adaptation [41]. Due to its many components, arbuscular mycorrhizal fungi (AMF) have been found to shift in response to shade conditions [42,43], suggesting their potential roles in plant adaptation to dim light environments. Finally, herbivorous insects represent an environmental component that was not assessed in this study. Shaded regions made by leaves may enhance the survival rate of herbivorous insects [44], reducing plant fitness, which might be responsible for selective pressure on shade adaptation.

### 3.3. Evolutionary Concerns Regarding the Climate Data Used in This Study

Our study conveyed how the various climate conditions could affect the physiological adaptation on the SAS in *B. juncea*. The climate data used in this study are based on the few decades, thus it might not be feasible to discuss the evolutionary time scale that spans up to million years ago. The cultivation of *B. juncea* has been documented at least 6000 to 7000 years ago [45]. Large-scale genomic analysis indicates *B. juncea* originates from 8000 to 14,000 years ago [2]. Considering these data, the time scale that should be considered in our study spans up tol 7000 years ago. This era is termed as the Holocene and spans from 10,000 years ago to the present. The mean surface temperature during the Holocene was shown to be very similar at the global level [46]. A study on summer monsoon precipitation shows that a somewhat comparable range was kept for the Holocene in terms of precipitation [47]. These long-term climate data could serve as mild evidence supporting our analysis. However, our analysis still has two huge limitations to overcome: (1) 7000 years is relatively short time span for considering the evolutionary change and (2) even though the climate data was expected to be comparable in large scale, the critical adaptational pressure could still remain in other conditions that we could not analyze in this study, such as soil composition or other evolutionary events. Limitation 1 can be overcome through a molecular study with critical gene sequences.

## 4. Materials and Methods

### 4.1. Plant Materials and SAS Analysis

Six cultivars (Akaoohadakana, Cheonggat, Dolsan, Dolsandaegyo, Eolcheong, and Jeokgat) were purchased from ASIA SEED Co., Ltd. (Seoul, Korea). Thirty clones comprising landraces and wild types were distributed from the National Agrobiodiversity Center, with the accession IDs indicated in Table S1. All seeds were sterilized with 70% ethanol for 5 min, rinsed with distilled water three times, and sown on a paper towel soaked with distilled water. Light source was FL20SSD/18 from WooriJomyung (Ansan, Korea). Then, the sown seeds were grown under continuous white light conditions (17.4827 W/m^2^) for 3 days and were either transferred to dim light (0.15326 W/m^2^) for 4 days or grown in white light conditions for 4 days to analyze the SAS phenotype.

### 4.2. Phenotype Analysis and Visualization

Hypocotyl lengths were measured for at least 10 fully grown seedlings with the ImageJ software v1.54g (http://imagej.nih.gov/ij/ accessed on 30 April 2025). For the visualization, the ggplot2 package from R was applied with options for violin plot and dot plot [48,49]. For the statistical analysis of hypocotyl length phenotype, the TukeyHSD test was performed using the aov and TukeyHSD functions in R [50]. To annotate the statistical significance, the multcompview function was applied to the statistical test results. Shade responsiveness was defined by the ratio of the hypocotyl length grown in dim light to the hypocotyl length grown in white light.

### 4.3. Climate Data Collection and Correlation Analysis

Based on the origin information of each wild type or landrace listed in Table S1, we collected the climate data of the natural origin. To minimize interannual variability, all climate variables were extracted for the same reference period (long-term climatological averages) for each origin site. Information on the temperature and precipitation of each site was collected from Climate Data (https://en.climate-data.org/ accessed on 26 March 2025). Daylength information was collected from the Astronomical Applications Department (https://aa.usno.navy.mil/data/Dur_OneYear accessed on 26 March 2025). Irradiation data was collected from the PHOTOVOLTAIC GEOGRAPHICAL INFORMATION SYSTEM (https://re.jrc.ec.europa.eu/pvg_tools/en/#PVP accessed on 26 March 2025). For the correlation analysis, latitude and longitude were normalized by adding 90 and dividing by 180. All the other climate data and phenotypes were normalized by subtracting the minimum value and dividing by the difference between the maximum value and minimum value. Then, all the data was normalized into a range of 0 to 1 by simple proportion and utilized for Pearson’s correlation analysis.

### 4.4. Machine Learning Practice and the Selection of the Appropriate Model

To identify the most precise machine learning model for predicting the phenotype by climate data, Orange3 was applied [51]. In detail, all the hypocotyl length data grown from white light or dim light was given to the machine, with corresponding climate data in separate input. Shade responsiveness was calculated from all the possible combinations and applied as well. Then, phenotype data was set as the target, and all the other data was set as the feature for machine learning models, including random forest, Adaptive boost (Adaboost), Support Vector Machine (SVM), k-Nearest Neighbor (kNN), Gradient Boosting, and Neural Network. To test each model, 20% of the total data was used for the test set. To address the performance of the models, we calculated RMSE (root mean square deviation) between predicted phenotypes and experimental data.

### 4.5. Random Forest Model and Feature Analysis

To identify partial contributions of each environmental factor, we made a random forest model of each phenotype (the hypocotyl length grown in white light, the hypocotyl length grown in dim light, or shade responsiveness predicted from climate data) in R, adapting ranger random forest model [52]. For the train data, 80% of the original data was used, while the other 20% was used as the test data. In building the random forest model, the number of trees was set as 1000, the importance was calculated with the permutation option, and the feature importance was calculated. For the permutation importance, the IML package was applied [53]. SHAP (SHapley Additive exPlanations) value was also calculated with the IML package with the SHapley function [53]. Partial dependence plots (PDP) were visualized using the IML package with the FeatureEffect function [53].

## 5. Conclusions

In this study, we analyzed shade avoidance syndrome (SAS) responses in *Brassica juncea* using six commercial cultivars and 30 landrace/wild-type clones from diverse geographic origins (one clone was excluded due to poor germination). All genotypes exhibited typical SAS traits under canopy-like (dim-light) conditions, including hypocotyl elongation and reduced cotyledon size, confirming a conserved shade-responsive program in *B. juncea*; however, the magnitude of hypocotyl elongation varied widely among genotypes, ranging from less than two-fold to more than seven-fold, indicating substantial intraspecific variation. Simple Pearson correlation analysis showed only weak associations between individual climatic variables and shade responsiveness and revealed strong intercorrelations among many climate factors, suggesting that single-factor linear analyses are insufficient to explain the observed variation. Therefore, we evaluated multiple machine learning models and identified random forest as the best-performing approach, demonstrating that hypocotyl phenotypes can be predicted from environmental variables and enabling estimation of feature contributions. The random forest models indicated that hypocotyl length under white light was mainly associated with latitude and minimum/maximum daylength, whereas hypocotyl length under dim light was more strongly influenced by precipitation- and temperature-related variables, along with irradiation metrics; notably, shade responsiveness was most strongly explained by maximum precipitation and maximum temperature, supporting the idea that combined climatic conditions at the site of origin contribute to variation in shade-adaptive hypocotyl plasticity in *B. juncea*.

## Data Availability

The original contributions presented in this study are included in the article/Supplementary Material. Further inquiries can be directed to the corresponding author.

## Supplementary Materials

The following supporting information can be downloaded at: https://www.mdpi.com/article/10.3390/plants15050780/s1, Figure S1: Machine learning model evaluation for predicting phenotypes based on climate data; Figure S2: Random forest model predictions of phenotypes based on climate data; Figure S3: Random forest model analysis of hypocotyl length under white light (WL) conditions with corresponding climate data; Figure S4: Random forest model analysis of hypocotyl length under dim light (DL) conditions with corresponding climate data; Table S1: List of analyzed clones or cultivars in this study; Table S2: Natural climate conditions of all the wildtypes or landraces in this study.

## Abbreviations

The following abbreviations are used in this manuscript:

bHLH	basic helix–loop–helix
SHAP	SHapley Additive exPlanations
