# Supplementary material for: Machine Learning Assessment of the Environmental Factors Contributing to Shade Adaptation in Brassica juncea
Source: Plants (Basel). 2026 Mar 3;15(5):780. doi: 10.3390/plants15050780 (PMC12986790; doi:10.3390/plants15050780)
Supplement: Supplementary file 1 [file plants-15-00780-s001.zip › plants-4171852-supplementary.pdf]

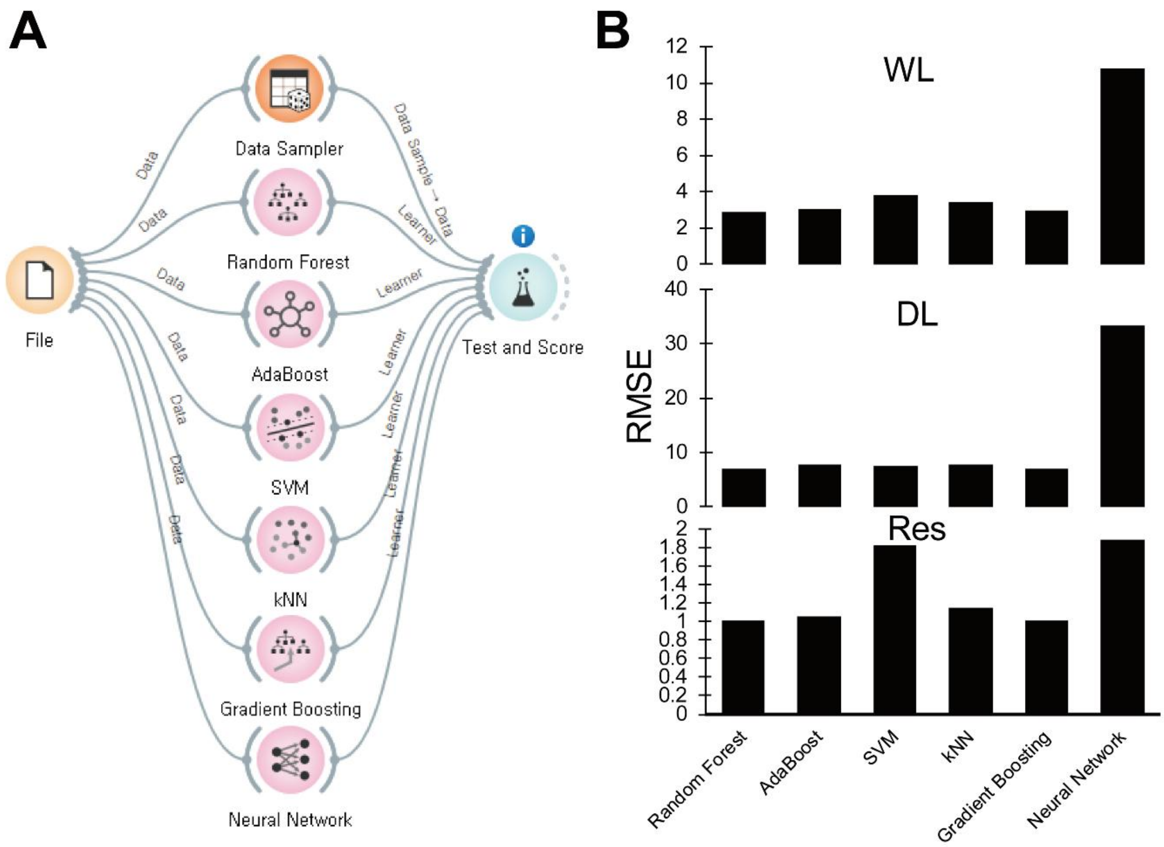

Figure S1. Machine learning model evaluation for predicting phenotypes based on climate data. (**A)** Orange3 pipeline used to identify the most accurate model for predicting the phenotypes based on climate data. The input file contained phenotype data with corresponding climate data. Tests were performed using hypocotyl length under white light (WL), dim light (DL), and shade responsiveness. (**B)** Bar graph depicting RMSE (Root Mean Squared Error) values from model test results.

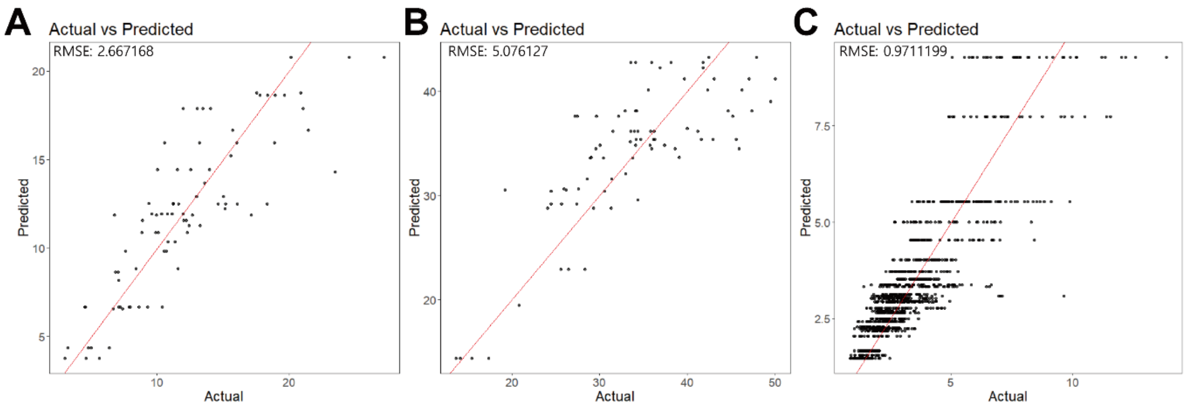

Figure S2. Random forest model predictions of phenotypes based on climate data. Actual indicates experimental data, while Predicted indicates model predictions. RMSE values are shown in the upper-left corner of each plot. (**A)** Comparison between predicted and experimentally measured hypocotyl length under white light (WL). (**B)** Comparison between predicted and experimentally measured hypocotyl length under dim light (DL). **(C)** Comparison between predicted and experimentally determined shade responsiveness.

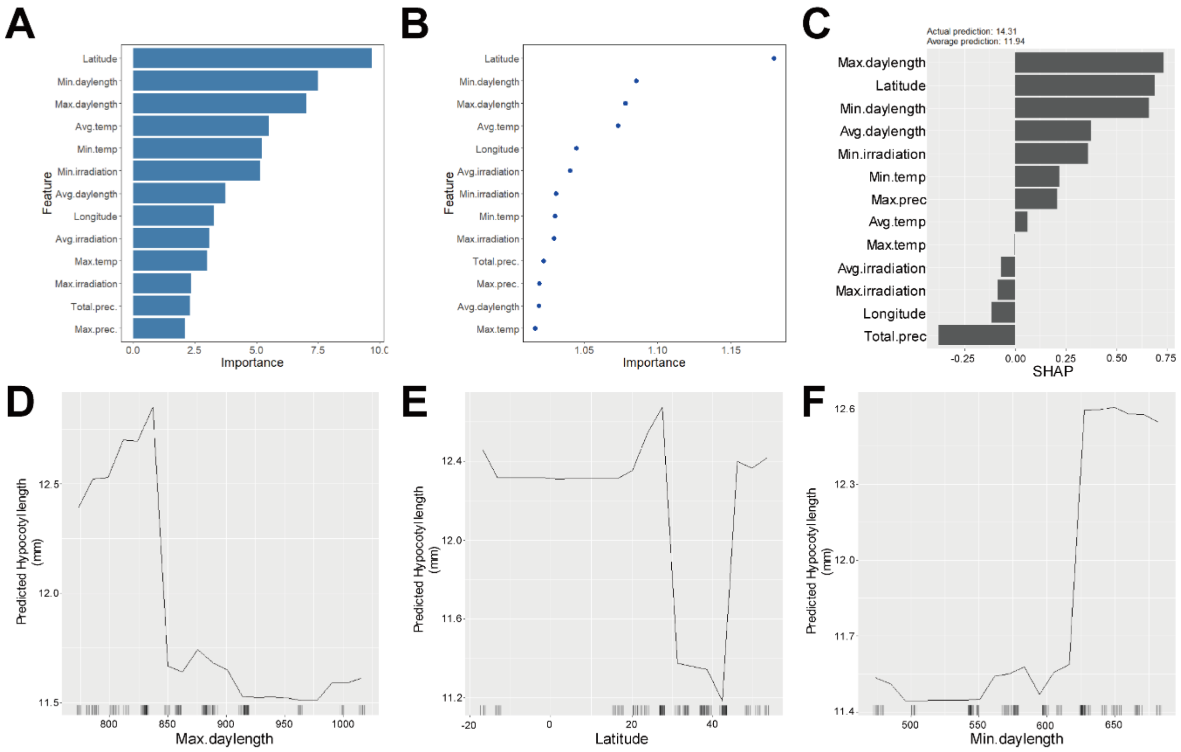

Figure S3. Random forest model analysis of hypocotyl length under white light (WL) conditions with corresponding climate data. (**A**) Bar graph representing feature importance in the model. (**B**) Dot graph representing feature importance determined by a permutation test. (**C**) Bar graph representing SHAP value. **(D-F)** Partial Dependence Plots of the three most influential features in predicting hypocotyl length under WL conditions: (**D**) maximum daylength vs. predicted hypocotyl length. (**E**) latitude vs. predicted hypocotyl length. (**F**). minimum daylength vs. predicted hypocotyl length.

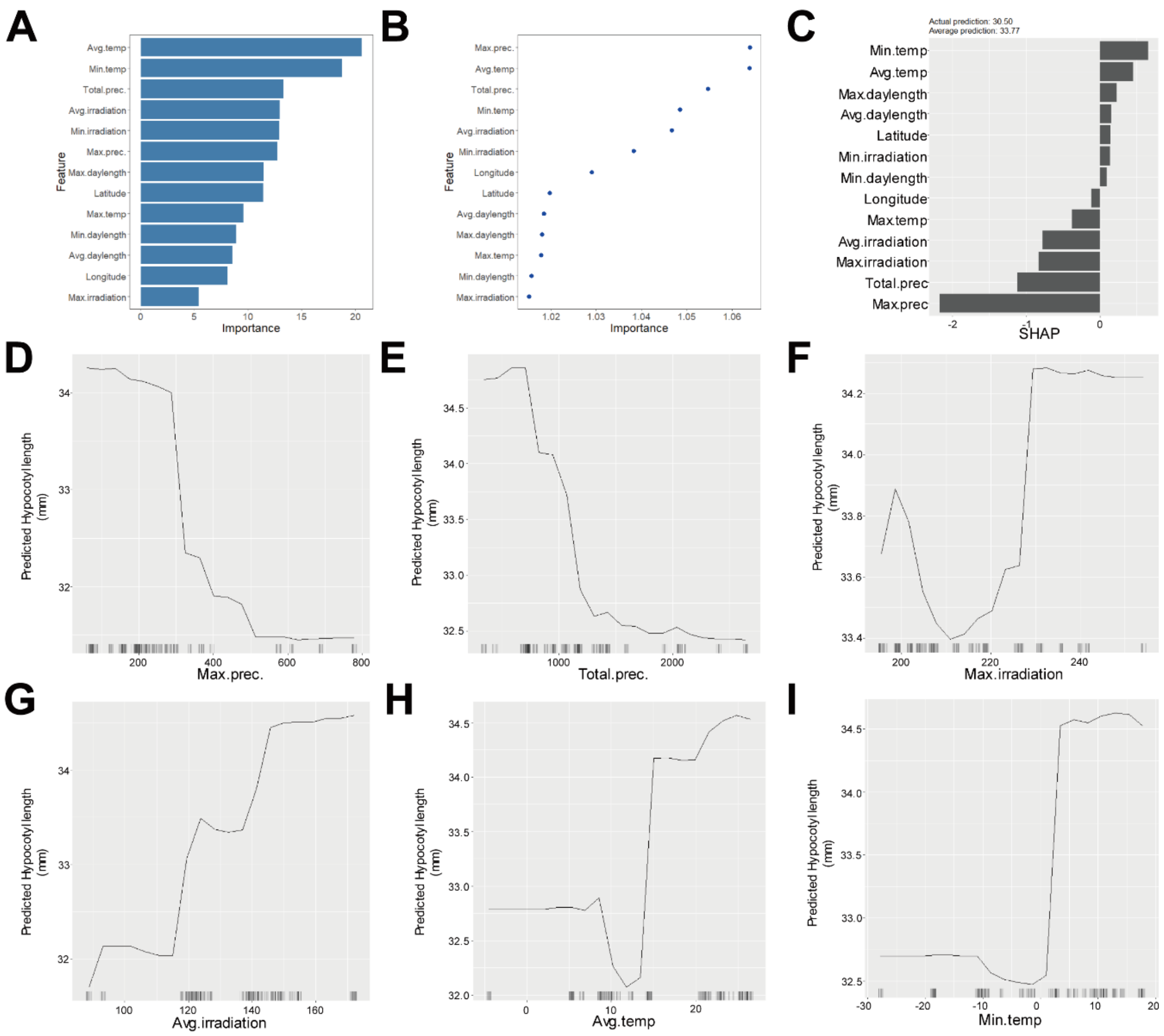

Figure S4. Random forest model analysis of hypocotyl length under dim light (DL) conditions with corresponding climate data. **(A)** Bar graph representing feature importance in the model. **(B)** Dot graph representing feature importance determined by a permutation test. **(C)** Bar graph representing SHAP value. **(D-I)** Partial Dependence Plots of the three most influential features in predicting hypocotyl length under DL conditions: **(D)** maximum precipitation vs. predicted hypocotyl length. **(E)** total precipitation vs. predicted hypocotyl length. **(F)** maximum irradiation vs. predicted hypocotyl length. **(G)** average irradiation vs. predicted hypocotyl length. **(H)** average temperature vs. predicted hypocotyl length. **(I)** minimum temperature vs. predicted hypocotyl length.

Supplemental Table S1. List of analyzed clones or cultivars in this study.

Longitude, Latitude	Accession	ID	Type
27.2569° N, 85.9713° E	IT136468	Bj01	Native
27.3709° N, 83.6667° E	IT136471	Bj02	Native
27.4310° N, 85.0320° E	IT136467	Bj03	Native
27.5065° N, 83.4377° E	IT136472	Bj04	Native
27.5291° N, 84.3542° E	IT136466	Bj05	Native
33.2524° N, 126.5126° E	IT228831	Bj06	Native
33.4111° N, 126.3939° E	IT216416	Bj07	Native
33.4111° N, 126.3939° E	IT293589	Bj08	Native
33.4946° N, 126.4590° E	IT109008	Bj09	Native
37.3677° N, 128.3954° E	IT101268	Bj10	Native
37.3797° N, 128.6636° E	IT195201	Bj11	Native
37.4441° N, 129.1679° E	IT103296	Bj12	Native
38.2107° N, 127.2165° E	IT217013	Bj13	Native
51.1657° N, 10.4515° E	IT314674	Bj14	Native
53.4785° N, 12.4217° E	IT279059	Bj15	Wild
48.5754° N, 89.5512° E	IT279064	Bj16	Wild
17.3342° N, 96.4778° E	IT250129	Bj17	Native
20.7888° N, 97.0337° E	IT248610	Bj18	Native
21.9588° N, 96.0891° E	IT271169	Bj19	Native
23.6850° N, 90.3563° E	IT306693	Bj20	Native
31.3260° N, 75.5762° E	IT278927	Bj21	Wild
13.0570° S, 27.5496° E	IT279037	Bj22	Wild
16.8621° S, 26.5138° E	IT279036	Bj23	Wild
42.8815° N, 129.3826° E	IT286636	Bj24	Native
42.8911° N, 129.5091° E	IT286637	Bj25	Native
42.8912° N, 129.5091° E	IT235357	Bj26	Native
42.3883° N, 77.2865° E	IT336241	Bj27	Native
42.8746° N, 74.5698° E	IT293386	Bj28	Native
15.8700° N, 100.9925° E	IT306679	Bj29	Native
38.9637° N, 35.2433° E	IT279198	Bj30	Wild
n.a.	Cheong-gat		Cultivar
n.a.	Jeok-gat		Cultivar
n.a.	Eolcheong-gat		Cultivar
n.a.	Dolsan-gat		Cultivar
n.a.	Dolsandaegyo-gat		Cultivar
n.a.	akaoohadakana		Cultivar

Description of types, wild = wild type, native = landrace, cultivar = cultivar

Supplemental Table S2. Natural climate conditions of all the wildtypes or landraces in this study.

ID	minimum temperature	maximum temperature	average temperature	maximum precipitation	total precipitation	minimum daylength	maximum daylength	average daylength	minimum irradiation	maximum irradiation	average irradiation
Bj01	7.7	29.6	21	612	1,930	627	829	729	89.83	211.95	136.8966
Bj02	9	39.8	25.3	299	939	626	830	729	87.89	226.69	146.6851
Bj03	4.9	23.4	21.6	778	2,100	626	830	729	85.56	216.96	141.3654
Bj04	9.6	35.6	24.2	394	1,366	626	830	729	91.04	226.49	144.6332
Bj05	9.4	32.6	23.2	690	2,407	626	831	729	79.46	218.76	138.869
Bj06	1.8	27.5	14.2	239	1,596	599	858	730	52.49	203.57	123.0508
Bj07	2.9	27.2	14.6	196	1,424	598	859	730	52.39	201.85	121.7102
Bj08	2.9	27.2	14.6	196	1,424	598	859	730	52.39	201.85	121.7102
Bj09	2.9	27.2	14.6	196	1,424	597	860	730	52.4	201.85	121.7359
Bj10	-10.4	25.4	8.7	257	1,163	577	881	731	56.63	206.57	124.334
Bj11	-10.9	25.4	8.7	222	1,180	577	881	731	53.76	207	123.6407
Bj12	-3.2	26.1	12.3	187	1,182	577	882	731	55.46	207.72	126.6679
Bj13	-9.9	27.5	10.1	335	1,310	572	886	731	51.53	205.19	118.2719
Bj14	-2.1	22.2	9	75	709	478	999	735	14.01	199.2	93.19772
Bj15	-1.4	22.3	9.4	83	723	474	1,016	736	11.59	199.14	88.68754
Bj16	-27.8	17.8	-4.5	71	336	502	963	734	43.46	235.7	139.3739
Bj17	17.8	37.2	26.5	575	2,645	666	788	728	74.77	215.59	142.5033
Bj18	10.8	29.9	20.6	202	1,032	653	802	728	101.61	218.75	148.7472
Bj19	14.3	37.4	26.3	197	1,071	649	806	728	108.55	212.35	149.468
Bj20	12.8	32.7	25.3	368	2,055	642	814	728	89.27	198.85	139.6618
Bj21	6	38.6	23.1	243	957	609	849	729	81.74	230.6	154.2878
Bj22	11.2	32.1	21.3	289	1,154	682	773	726	117.64	240.03	171.6115
Bj23	10.7	34.5	22.7	215	788	668	787	726	109.46	241.4	172.1445
Bj24	-18.6	25.6	5.3	160	720	544	917	732	58.03	196.34	120.9848
Bj25	-18.6	25.6	5.3	160	720	544	917	732	58.04	195.41	120.1054
Bj26	-18.6	25.6	5.3	160	720	544	917	732	58.04	195.41	120.1054
Bj27	-10	23.8	6.5	150	891	548	913	732	55.14	225.71	141.3039
Bj28	-9.3	28.9	9.8	126	682	544	917	732	62.61	231.11	146.5889
Bj29	17.4	34.1	26	273	1,367	630	781	728	98.47	204.1	154.6959
Bj30	-6.7	30	10.9	61	433	569	890	731	58.28	254.19	152.3952

Temperature is annotated with degree in Celsius, precipitation is annotated with millimeter. Maximum precipitation is annotated per month. Daylengths are in minutes and irradiation is in kWh/m^2.
